# Validation of the osteoporosis quality of life questionnaire QUALEFFO-41 for the Serbian population

**DOI:** 10.1186/1477-7525-10-74

**Published:** 2012-06-18

**Authors:** Ivana Tadic, Nada Vujasinovic Stupar, Ljiljana Tasic, Dejan Stevanovic, Aleksandar Dimic, Bojana Stamenkovic, Sonja Stojanovic, Sasa Milenkovic

**Affiliations:** 1 Department of Social Pharmacy and Pharmaceutical Legislation, University of Belgrade, Faculty of Pharmacy, Vojvode Stepe 450, 11000, Belgrade, Serbia; 2 University of Belgrade, School of Medicine, Institute of Rheumatology, Belgrade, Serbia; 3 Department of Social Pharmacy and Pharmaceutical Legislation, University of Belgrade, Faculty of Pharmacy, Belgrade, Serbia; 4 Department of Psychiatry, General Hospital Sombor, Sombor, Serbia; 5 Rheumatology Institute Niska Banja, University of Nis, Faculty of Medicine, Nis, Serbia; 6 Rheumatology Institute, Niska Banja, Nis, Serbia

**Keywords:** Osteoporosis, Validation, Quality of life

## Abstract

**Background:**

Vertebral fractures could lead to reduced physical, social and mental functioning, and loss of personal independence. Therefore, during the treatment of osteoporosis, it has become necessary to examine the changes in everyday functioning, well-being and health related quality of life (HRQOL). To that effect, this study aims to translate, culturally adapt, and validate the Serbian version of Quality of Life Questionnaire of the European Foundation for Osteoporosis (QUALEFFO-41) for patients with vertebral fractures.

**Methods:**

Nine female patients with osteoporosis participated in the pre-validation study. A validation, case–control study included two groups of female patients: one that consisted of 50 female patients with osteoporosis, and with at least one vertebral fracture, and another one that consisted of 50 control patients with osteoporosis but without fractures. They completed the QUALEFFO-41 and the EuroQol group questionnaire with five dimensions (EQ-5D) twice within a month. The validation study examined internal consistency, concurrent validity, test-retest reliability, sensitivity and specificity.

**Results:**

During the pre-validation study, three of the items in the QUALEFFO-41 were slightly changed. Afterwards, during the validation study, the statistically significant differences (adjusted for: age, duration of menopause, current employment and marital status) in the mean values of all domains and total scores between the groups were noted. For the case group, the internal consistency of the QUALEFFO-41 domains and of total questionnaire was above 0.70. The test-retest reliability was tested by the intraclass correlation coefficients (ICC) that were in range 0.87 – 0.96 for the case, and 0.15 – 0.83 for the control group. Correlations between the total scores of the QUALEFFO-41 and the EQ-5D health state value, for both groups were negative and statistically significant (r = -0.78, p<0.001 and r = -0.73, p<0.001, respectively). The QUALEFFO-41 had a better prediction of the value of HRQOL of cases compared to the generic questionnaire EQ-5D (the AUC difference was 0.099, p = 0.013).

**Conclusions:**

The Serbian QUALEFFO-41 version is reliable, valid, sensitive and predictive for examinations of HRQOL in patients with prevalent vertebral fractures and can be used in further studies.

## Background

The most common fractures in osteoporosis are the vertebral ones [1]. If at least one vertebral fracture exists, the risk of the new ones is fivefold higher during the initial year [2]. These fractures can cause back pain, kyphosis, difficulties in performing daily activities, depression and anxiety, all of which can be reflected in physical, social and mental functioning and loss of personal independence [3-7]. Severity of these symptoms is related to the degree of deformity and the number of fractures. In average, back pain remains almost the same five years after the diagnosis of the vertebral fractures has been established, while key physical functions of independent living could deteriorate [4]. The treatment progress of patients with vertebral fractures should not be based only on the clinical outcomes, but on the humanistic ones as well, such as the health related quality of life (HRQOL) outcome.

According to the literature, the Quality of Life Questionnaire of the European Foundation for Osteoporosis (QUALEFFO-41) is the most frequently used questionnaire for measuring the HRQOL of patients with osteoporosis and vertebral fractures [8-15]. The QUALEFFO-41 is specific, sensitive, and questionnaire which are able to discriminate between patients with and without vertebral fractures [16]. It is shown that the questionnaire is sensitive to the number of fractures. The QUALEFFO-41 score decreases with increasing number of vertebral fractures [5,9].

This questionnaire covers main aspects of quality of life: pain, physical functions, social functions, general health and mental health [15]. There are several versions of the questionnaire: the first one had 48 items and it was shortened to 41 items [17]. A short version, with 31 items, also exists [18]. The QUALEFFO-41 is still the most applied questionnaire in different studies and cultures [7,15,17-21].

There has been no previously validated specific HRQOL questionnaire for osteoporosis with vertebral fractures in Serbia. Therefore, the aim of this study is to examine psychometric properties and validate the Serbian version of QUALEFFO-41. As we cannot compare the results of this one to other specific questionnaires, a generic questionnaire the EuroQol group questionnaire with five dimensions (EQ-5D) previously translated and culturally adapted for Serbian population was used [22].

## Methods

This is a case–control study conducted during the period of June 2010 - October 2011. The study was performed in two medical centres: The Institute of Rheumatology - Medical Faculty, University of Belgrade and The Institute for Prevention and Treatment of Rheumatic Diseases “Niska Banja”, Medical Faculty University of Nis. The study was approved by the Ethics Committees of both institutes.

All patients were recruited by their physicians. Patients who agreed to participate provided a written informed consent before enrolment. The QUALEFFO-41 and the EQ-5D were administered within one month. The questionnaires have always been administrated in the same order: QUALEFFO-41 followed by the EQ-5D. First, patients completed a set of the questionnaires during regular clinical visits without help from the medical staff or family members. At this time, one more set of the questionnaires was given to every patient. Afterwards, approximately one month later, they were reminded by phone to complete the set of the questionnaires again, and to send them back by mail to the main researcher (T.I.).

### Participants

The patients with osteoporosis participated in the pre-validation study (translation and cultural adaptation of the QUALEFFO-41 questionnaire). Responses of these patients were not considered in the validation study.

The validation study was conducted with patients suffering from osteoporosis with at least one vertebral fracture (case group), and the control group of patients with osteoporosis and with no fractures. Main inclusion criteria for both groups were the same: postmenopausal women over 45 years of age, with primary osteoporosis, who were able to read and write Serbian language. The following exclusion criteria were also applied: presence of chronic back pain caused by other diseases, inflammatory rheumatic disease, malignant or metabolic bone diseases, and usage of glucocorticoids. Patients with incidental vertebral fractures during the one month period prior to the study were excluded.

Sample size was calculated as a number of patients needed for paired *t*-test (to test the difference in results between the case and the control group). For α = 0.05 and 80% power of detection 33 patients in each group are needed [23]. This study also tested reproducibility of the questionnaire. A sample size, needed to detect the differences in patients’ agreements, had to be at least 50 [24]. Therefore, up to 50 eligible patients meeting the criteria were included for each group.

### Diagnosis of osteoporosis and vertebral deformity measurement

Primary osteoporosis was diagnosed by Dual-energy X-Ray Absorptiometry (DXA) measurement on the lumbar spine (L1-L4) and/or the hip. The DXA measurement was performed using the GE Lunar Prodigy, according to the World Health Organization criteria [25]. Fractures were defined according to the Genant's classification (vertebral anterior, middle or posterior height reduction more that 20%) [1]. Vertebral deformities were established by the lateral radiography of the thoracic and lumbar regions (T4-L5) by an experienced radiologist.

### Translation and cultural adaptation of QUALEFFO-41 questionnaire

**Figure 1:**
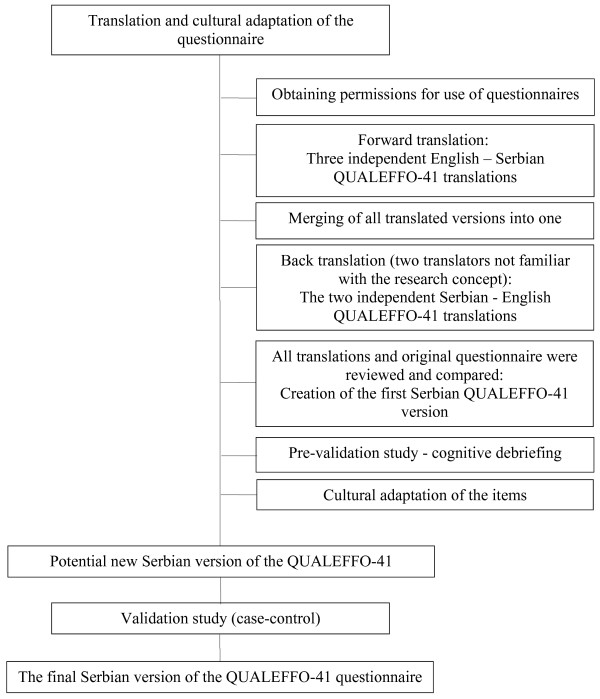
The translation and cultural adaptation process of the QUALEFFO-41 questionnaire.

The translation and the cultural adaptation of the QUALEFFO-41 were performed according to the International Society for Pharmacoeconomics and Outcomes Research guidelines [26]. First, permissions to use the questionnaires were obtained from the copyright holders (for the EQ-5D-Serbian version from the EuroQol group and for the QUALEFFO-41 from the International Osteoporosis Foundation). Then, three authors T.I., S.D. and V.S.N. participated in the forward translation process and made three independent English – Serbian QUALEFFO-41 translations (“T1”, “T2” and “T3”). All translators (native Serbian speakers) were familiar with the topic and the research concept. Through discussions between them, the new version “T123” was created as a result of merging all of the translated versions. In the process of back translating, two independent translators, who were not familiar with the research concept, translated version T123 back into English (versions “B1” and “B2”). The original QUALEFFO-41 questionnaire, the forward and backward translated versions, were reviewed and compared in order to check the conceptual equivalence. During the last phase (cognitive debriefing), the questionnaire was pre-tested on a sample of osteoporosis patients. This group of patients was chosen irrespectively of presence of vertebral fractures. After the patients had completed the questionnaire, they were asked to comment on the simplicity, clarity and relevance of the items. Translation, cultural adaptation and validation steps were summarized on the Figure 1.

### Questionnaires

The QUALEFFO-41 is a self-administered, disease specific questionnaire [15]. Most of the items were developed by the clinicians of the Working Party of the European Foundation for Osteoporosis and two were taken from the Mediterranean Osteoporosis Study (MEDOS) Questionnaire and European Vertebral Osteoporosis Study (EVOS) Questionnaire [17]. It possesses five domains (41 items in total): pain (5 items), physical function (17 items), social function (7 items), general health perception (3 items) and mental function (9 items). Most of the items have 5 answer options. Exceptions have been made for the items No 23, 24, 25, and 26 with 3, and items No 27, 28, and 29 with 4 answer options. Items are scored in the reverse order (the minimum number on the scale is assigned to the best answer and the maximum to the worst answer), except for the items No 33, 34, 35, 37, 39 and 40. The score of each domain is calculated as an average value of all the answered items linearly transformed on a scale 0-100. The total QALEFFO score is calculated as a sum of all answers to items and then linearly transformed on the scale 0-100. The worse the HRQOL condition is, the higher the score gets. The scoring algorithm is made in a way to calculate the total score proportionally to the answered items (when a missing value is present, the calculations are corrected according to the number of missing values). According to the scoring algorithm the missing value should not have exceeded 30%, otherwise the domain or the total score becomes inaccurate.

The EQ-5D is a self-administered, general questionnaire and can be used in various population groups. The EQ-5D consists of two parts: a self-reported 5-item questionnaire and a visual analogue scale (VAS). All items have 3 answer options. The EQ-5D health state value (HSV) is calculated according to the user manual [27,28]. The EQ-5D HSV ranges from 0 (the worst possible HRQOL condition) to 1 (the best possible HRQOL condition). The VAS is a 100 degree thermometer - like scale that represents the current health state. The VAS score is defined as the value that patients mark on the scale at the place that reflects their current health state the best.

### Statistical analysis

Patients’ demographics and clinical data were collected during the inclusion of patients. They included: age, age at menopause onset, employment and marital status, education level, number of fractures and fracture site. The descriptive analysis included calculation of mean, SD, distribution of missing data, floor and ceiling effects. This analysis was performed for both groups, and it was calculated for all domains and total scores. The ceiling effects represent a percentage of the highest possible scores, opposite to the floor effects. The presence of high values of the floor or ceiling effects could indicate poor discrimination of items and thus reduced sensitivity of the questionnaire [29]. The floor and ceiling effects should be less than 15%, if a scale encompasses the entire range of possible responses [24]. Differences between the demographic characteristics of groups were tested using *t*-test. Differences between the mean values of the domain scores of the groups were tested using ANCOVA. The test was adjusted for all the variables that were shown to be significantly different between the groups.

Internal consistency was assessed using the Cronbach’s α coefficient. The Cronbach’s α value should be greater than 0.7 for a scale of good consistency [30].

Pearson’s correlation coefficient was calculated between all the items and their domain score to check item’s convergent validity. The correlation coefficient should be moderate to large (≥0.4). A discriminant validity checks if the item measures other concept that it is not supposed to measure. A degree of item’s discriminant validity was calculated as a correlation between items and scores of other domains. The discriminant validity of each item should be lower than the convergent one [29].

Reproducibility was estimated through a test-retest after one month. The strength of agreement between the repeated measures was examined using the intraclass correlation coefficient (ICC). The recommended value of ICC is ≥0.7, but it is considered as acceptable if it is ≥ 0.6 [29].

Concurrent validity was explored by correlations between the scores of the QUALEFFO-41 and EQ-5D questionnaires. Since the EQ-5D has only five items, a correlation coefficient was calculated only between the total score of the QUALEFFO-41 and the EQ-5D HSV.

Finally, the sensitivity was assessed by the Receiver Operating Characteristics Curve (ROC) analysis. This analysis can describe discrimination ability of a questionnaire. The ROC is defined by the values of sensitivity (true positive rate) and specificity (true negative rate) of the questionnaire. Sensitivity is an ability of a questionnaire to detect a patient with the observed disease versus specificity (that is the ability to detect a patient without the disease). This analysis included comparison of the areas under the ROC curves (AUC) over all possible cut-off values of the questionnaire’s scores [15,31,32]. A sensitive questionnaire should have AUC values above ≥ 0.7 [24].

Statistical analyses were performed using Statistical Package for Social Sciences (SPSS) version 18.0 and MedCalc package version 11.6.1.

## Results

Nine female patents participated in the pre-validation study. Two of them were with osteoporosis and with vertebral fractures. The average age of the patients was 64.89 years (SD = 3.48).

No item was replaced or omitted from the QUALEFFO-41-The Serbian Version in the pre-validation study. According to the patients’ opinions, most of the items were understandable, clear, precise, and relevant to the HRQOL assessments. Only three of the items were slightly changed. In the item No 26 (“Can you visit a cinema, theatre, etc.?”) one more answer (“No financial means for that activity”) was added in accordance with patients’ opinions. This change did not have an effect on the scoring of the social function domain. In the item No 14 (“Can you lift a heavy object of 20 lbs (e.g. a crate of 12 bottles of milk, or a one year old child), and carry it for at least 10 yards?”) Anglo-Saxon units were converted into the International System of Units (SI) (lbs into kg, and yards into metres). The item No 18 was slightly changed in accordance with the living conditions in Serbia (“Can you climb stairs to the next floor of a house” was replaced with “Can you climb stairs to the next floor”). This replacement did not affect the meaning of the item, and the item still conceptually corresponds to the physical function domain.

### Subjects

In the validation study, 110 female patients were recruited (52 cases and 58 controls). Two of the patients in the case group did not meet the inclusion criteria (response rate for the case group was 96.15%). Seven of the patients in control group did not return the questionnaires at the initial recruitment, and one of them did not meet the inclusion criteria (response rate for the control group was 86.21%). After one month, 80% of cases and 52% of controls completed and sent-back the set of questionnaires. Average time between the first and the second assessment was 30.40 days (SD = 9.51) for the cases and 32.48 days (SD = 11.29) for the controls. At the initial assessment, it took patients approximately fifteen minutes to complete the set of questionnaires.

In average 2.12 (SD = 1.70, range 1-9) vertebral fractures were noted in the case group. Patients usually had one vertebral fracture (55.10%) and the most frequent fracture was located at the L1 vertebra (24.04%). Information about the number and the site of fractures are provided in additional files [see Additional file 1] [see Additional file 2].

Significant differences between the groups were found regarding the age, the duration of menopause, the employment, and the marital status (Table 1). These variables were included as the controlling ones in the analyses below.

**Table 1:** Socio-demographic and clinical characteristics of the case and control subjects

	**Cases (n = 50)**	**Controls (n = 50)**	**p-value**
Age mean in years (SD)	71.84 (8.57)	63.02 (7.49)	p < 0.001
Mean age of menopause onset (SD)	47.10 (5.13)	47.02 (5.36)	0.93
Duration of menopause in years (SD)	24.74 (8.98)	16.00 (9.62)	p < 0.001
BMI mean (kg/m^2^) (SD)	25.14 (4.53)	25.71 (3.76)	0.50
Employment status (N, (%))			0.01
Working	1 (2.00)	7 (14.00)	
Not working	5 (10.00)	10 (20.00)	
Medical leave of absence	2 (4.00)	0 (0.00)	
Retired	42 (84.00)	33 (66.00)	
Education level (N, (%))			0.62
No formal education	0 (0.00)	2 (4.00)	
Primary	18 (36.73)	15 (30.00)	
Secondary	11 (22.45)	22 (44.00)	
Master degree	18 (36.73)	10 (20.00)	
Postgraduate	2 (4.08)	1 (2.00)	
Marital status (N, (%))			p < 0.001
Married	18 (36.00)	38 (76.00)	
Single (including divorced)	8 (16.00)	3 (6.00)	
Widowed	24 (48.00)	9 (18.00)	

*SD*, Standard Deviation

*BMI*, Body Mass Index

### Descriptive statistics

Between the groups, the differences (adjusted for the age, the duration of the menopause, the current employment and the marital status) in the mean values for all domains and total scores were statistically significant.

The number of missing values for both groups was below 5.2%. An exception in the case group was noted for the social function domain (22%).

A floor effect above 15% was observed for the general perception domain in both groups. High percentage value (30%) of ceiling effect was noted in control group for the pain domain. The difference in VAS scale between the groups was consistent with the difference of HSV. But, the difference of VAS mean values was not statistically significant (p > 0.05) (Table 2).

**Table 2:** Descriptive statistics of the QUALEFFO-41 and EQ-5D questionnaires

**Questionnaire domain**	**Mean (SD)**		**Missing values inside domain (%)**		**Floor (%)**		**Ceiling (%)**	
	**Controls**	**Cases**	**Controls**	**Cases**	**Controls**	**Cases**	**Controls**	**Cases**
QUALEFFO-41								
Pain	40.78 (32.58)	55.20^*^ (26.22)	13 (5.20)	9 (3.60)	2.00	6.00	30.00	8.00
Physical function	23.65 (18.64)	42.56^**^ (22.26)	10 (1.18)	14 (1.65)	4.00	4.00	10.00	2.00
Social function	52.15 (23.16)	67.70^**^ (23.47)	10 (1.18)	77 (22.00)	8.00	6.00	2.00	2.00
General health perception	64.08 (19.69)	74.33^*^ (21.68)	2 (1.33)	1 (0.67)	16.00	24.00	2.00	2.00
Mental function	28.40 (14.59)	38.10^*^ (15.66)	4 (0.89)	8 (1.78)	2.00	2.00	2.00	4.00
Total QUALEFFO-41 score	35.71 (16.73)	51.86^*^ (19.00)						
EQ-5D								
HSV	0.58 (0.20)	0.46^*^ (0.28)	10 (4.00)	10 (4.00)				
VAS scale	50.46 (23.21)	48.60 (21.54)	4 (8.00)	2 (4.00)				

^*^P<0.05, ^**^P<0.001, ANCOVA

*SD*, Standard Deviation

*QUALEFFO-41*, Quality of Life Questionnaire of the European Foundation for Osteoporosis

*EQ-5D*, EuroQol Group Questionnaire

*HSV*, Health State Value

*VAS*, Visual Analogue Scale

### The QUALEFFO-41 and EQ-5D

The Cronbach’s α value for the QUALEFFO-41 domains ranged from 0.79 to 0.93 for the case and from 0.33 to 0.92 for the control group. Cronbach’s α for the EQ-5D was 0.84 for the case and 0.75 for the control group.

All the items correlated better with their own domain score, than with the other domain scores.

Test-retest reliability in both groups was satisfactory with all ICC values above 0.6, except for general health perception domain in the control group (Table 3).

**Table 3:** Results of the multi-trait statistics of QUALEFO-41 domains and EQ-5D

**Questionnaire/domain**	**Cronbach's α (range of Cronbach's α if item deleted)**		**Range of item correlation coefficient with own domain (%)^a^**		**Range of item correlation coefficient with other domains (%)^a^**		**Intraclass correlation coefficient (n = 38)**	
	**Controls**	**Cases**	**Controls**	**Cases**	**Controls**	**Cases**	**Controls**	**Cases**
QUALEFFO-41								
Pain	0.82 (0.73-0.87)	0.86 (0.81-0.86)	0.21-0.92 (80.00)	0.69-0.88 (100.00)	-0.21-0.47 (20.00)	0.15-0.55 (60.00)	0.78 (0.53-0.90)	0.87 (0.76-0.87)
Physical function	0.92 (0.91-0.93)	0.93 (0.92-0.94)	0.33-0.84 (94.12)	0.32-0.86 (94.11)	0.03-0.69 (45.59)	0.01-0.66 (76.47)	0.83 (0.62 - 0.92)	0.94 (0.88-0.97)
Social function	0.33 (0.08-0.51)	0.80 (0.75-0.80)	0.03-0.71 (57.14)	0.46-0.81 (100.00)	-0.29-0.60 (17.86)	-0.10-0.74 (50.00)	0.78 (0.52 - 0.90)	0.91 (0.82-0.95)
General health perception	0.48 (0.12-0.72)	0.81 (0.62-0.89)	0.45-0.83 (100.00)	0.77-0.91 (100.00)	-0.01-0.45 (25.00)	0.38-0.73 (91.67)	0.15 (-0.59 - 0.55)	0.91 (0.84-0.95)
Mental function	0.74 (0.66-0.75)	0.79 (0.74-0.79)	0.39-0.79 (55.56)	0.37-0.81 (66.67)	-0.01-0.62 (27.78)	0.01-0.65 (30.56)	0.76 (0.47 - 0.89)	0.89 (0.80-0.94)
Total QUALEFFO-41 score	0.91 (0.90-0.92)	0.83 (0.75-0.89)					0.82 (0.60 - 0.92)	0.96 (0.92 - 0.98)
EQ-5D								
Health state value	0.75 (0.73-0.88)	0.84 (0.83-0.89)	-0.77- -0.56 (100.00)	-0.82- -0.66 (100.00)				

^a^A percent of correlation coefficients higher than 0.4

*QUALEFFO-41*, Quality of Life Questionnaire of the European Foundation for Osteoporosis

*EQ-5D*, EuroQol Group Questionnaire

A correlation between the total score of the QUALEFFO-41 and EQ-5D HSV was negative and statistically significant (Spearman’s rank correlation coefficients for cases and controls were r = -0.78, p<0.001 and r = -0.73, p<0.001, respectively).

**Figure 2:**
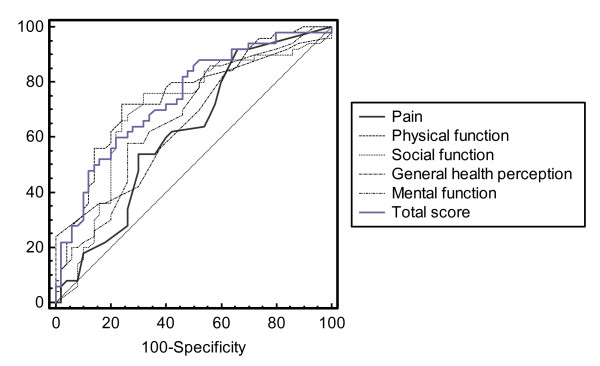
ROC curve for QUALEFFO-41 domains and total score. Discrimination between cases and controls.

The ROC curve analysis indicated a modest ability of QUALEFFO-41 to detect the difference in HRQOL scores between the case group of patients and the control ones (Figure 2).

The AUC of the QUALEFFO-41 domains and the total score showed poor discriminatory capacity (AUC range 0.62 – 0.69). However, the QUALEFFO-41, compared to EQ-5D, could better predict the value of HRQOL in patients with vertebral fractures (difference between areas was statistically significant for p<0.05) (Table 4).

**Table 4:** ROC curve analysis of questionnaires QUALEFFO-41 and EQ-5D

	**QUALEFFO- 41 area (SE)**	**CI**	**EQ-5D HSV area (SE)**	**CI**	**Difference area (SE)**	**p- value**
Pain	0.62 (0.056)	0.52 - 0.72				
Physical function	0.75 (0.049)	0.65 - 0.83				
Social function	0.69 (0.052)	0.59 - 0.78				
General health perception	0.65 (0.055)	0.55 - 0.74				
Mental function	0.67 (0.054)	0.57 - 0.76				
Total QUALEFFO- 41 Score	0.74 (0.050)	0.65 - 0.82	0.64 (0.056)	0.54 - 0.74	0.099 (0.040)	0.013

*ROC*, Receiver Operating Characteristics

*QUALEFFO-41*, Quality of Life Questionnaire of the European Foundation for Osteoporosis

*EQ-5D*, EuroQol Group Questionnaire

*HSV*, Health State Value

*SE*, Standard Error

*CI*, Confidence Interval

## Discussion

This is the first study in Serbia in regards to reporting on the validation of the osteoporosis specific HRQOL questionnaire, the QUALEFFO-41. The results indicate that the Serbian version of the QUALEFFO-41 has satisfactory general psychometric characteristics.

The QUALEFFO-41 was well accepted by patients who participated in the pre-validation study. Most of the items, in accordance with the patients’ opinions, were simple, clear, and relevant to the HRQOL assessment, and only three items underwent slight changes. In the item No 26 “Can you visit a cinema, theatre, etc.?” the answer “No financial means for that activity” was added. This change is related to the living condition in Serbia, where most of the retiree patients have had an average retirement income of approximately 220EUR per month. Changes in two more items (No 14 and No 18) were in relation to the SI metric unites and the living conditions in Serbia. None of the aforementioned changes affected the meaning of the items, the domains concepts and the scoring of QUALEFFO-41.

The results of the general analysis showed the following. The results of the missing value analysis indicated that the most of the QUALEFFO-41 domains possess good acceptability. However, the highest missing value was found in the group of patients with vertebral fractures regarding the social function domain. A possible explanation could be related to the disability of patients to engage in the sports activities, do the gardening, and go to the cinema/theatre. Furthermore, the menopausal women found the item: “Does your back pain or disability interfere with intimacy (including sexual activity)?”too private to answer. This item, also a part of the social function domain, could also be related to the level of disability caused by vertebral fractures.

The floor and ceiling effects were acceptable in both groups of patients. Only the general health perception domain had a high floor effect in the group of patients with fractures, and the pain domain had a high ceiling effect in the group of patients without the fractures. The patients with fractures usually have pain and physical disabilities that can influence their general health perception as of being poor. This is in accordance with the fact that the osteoporosis can be asymptomatic until the fractures occur [33]. The patients without fractures haven’t had any back pain and thus more readily choose more favourable responses in the pain domain. Nevertheless, these results could indicate that these two domains have a poor discrimination and thus a reduced sensitivity and responsiveness.

The mean QUALEFFO-41 values were higher in the patients with fractures than in the control group, as opposed to the results of the EQ-5D HSV. Such a result was expected, because the scoring of these two scales went in the opposite directions. These results indicated that the patients with fractures had poorer levels of HRQOL than the patients without fractures. These results are in accordance with a similar study [21].

Considering the psychometric properties of the Serbian version of QUALEFFO-41, several important facts were observed. In the group of patients with fractures, the values of Cronbach’s α in all the QUALEFFO-41 domains were higher than 0.70, indicating a good internal consistency. Similar Cronbach’s α values were reported regarding other language versions, e.g.: Arabic (range 0.74-0.89), Turkish (range 0.70-0.90) and some European versions (range 0.72-0.92) [15,20,21]. For the control group of patients, the internal consistency was also satisfactory regarding the overall questionnaire and some of the domains, but in regards to the social function and general health perception domains, it was below 0.7. The study conducted on the Mexican population reported a low Cronbach’s α value in the social domain, even in the case group of patients (patients with vertebral fractures). This study had tested both, the social function domain including all seven items, as well as the one excluding two items, and it showed that a shorter version of the domain was more acceptable to be the part of the questionnaire (Cronbach’s α value was 0.46 in the seven-item domain and 0.71 in the five-item domain)[19].

Convergent validity was satisfactory for the group of patients with fractures, with two exceptions: the low correlation coefficient values were noted in the domains of the physical and mental functions. Considering the group of patients without fractures, only in the domain of the general health perception, satisfactory correlation coefficient values, have been observed.

In both groups, the items were more convergent than discriminant. These results indicated a good distribution of items in the domains. According to these facts, the items measured the same concept in each domain.

The strength of agreement between repeated measures in the group of patients with fractures was satisfactory for all domains. In the control group of patients, the ICC values for the general health perception domain were below the acceptable value. In addition, this domain has had a low internal consistency. It can be assumed that this domain is not suitable to be a part of the questionnaire for the osteoporosis patients without fractures.

A concurrent validity was explored by correlations between QUALEFFO-41 and EQ-5D questionnaires and the results showed that the questionnaires were highly correlated and both measured the HRQOL aspects.

The AUC values indicated that QUALEFFO-41 had a low to moderate power to discriminate HRQOL domain scores of patients with and without vertebral fractures. The study conducted in Mexico with the same type of case–control patients has shown similar results (vales of the AUC were in range from 0.52 to 0.73) [19]. Lips et al. also reported equivalent results (values of the AUC were in range from 0.64 to 0.78), for patients with vertebral fractures as a case group and healthy controls [15]. We expected to get lower AUC values compared to the study reported by Lips et al., taking into account that we included patients with lesser differences in health conditions between the groups.

The AUC value of QUALEFFO-41 total score was higher and significantly different than that of the EQ-5D. Therefore, the QUALEFFO-41 has more power to predict the HRQOL in patients with vertebral fractures in relation to the EQ-5D.

We have noted several limitations in our study. First, we did not use another specific HRQOL questionnaire to evaluate the concurrent validity, since there had been no such previously validated questionnaire with similar domains in Serbia. Second, we used a uni-dimensional generic questionnaire EQ-5D, so that we could make comparisons only between the total scores of questionnaires as an external criterion. Third, we did not compare QUALEFFO-41 scores of patients with different numbers of vertebral fractures. Thus, the sensibility of the questionnaire to detect differences in the HRQOL, according to the number of vertebral fractures was left unexplored. Finally, the construct validity was not evaluating using a factor analysis, either explorative or confirmatory, considering the small number of patients. Further research should include larger number of patients, which would allow us to use the factor analysis, and thus complete the validation of the questionnaire.

## Conclusions

This study has shown that the Serbian version of the specific osteoporosis questionnaire, QUALEFFO-41, has been well-accepted by the patients. The questionnaire has an appropriate internal consistency, test-retest reliability, sensitivity and specificity. Thus, the Serbian QUALEFFO-41 version possesses satisfactory general psychometric characteristics and it can be used in clinical studies on patients with vertebral fractures.

## Abbreviations

HRQOL, Health Related Quality of Life; QUALEFFO-41, Quality of Life Questionnaire of the European Foundation for Osteoporosis; EQ-5D, EuroQol Group; ICC, The Intraclass Correlation Coefficient; DXA, Dual-energy X-Ray Absorptiometry; VAS, Visual Analogue Scale; HSV, EQ-5D Health State Value; ANCOVA, Analysis of Covariance; ROC, Receiver Operating Characteristics Curve; AUC, Area Under the Curve; SPSS, Statistical Package for the Social Sciences.

## Competing interests

The authors declare that they have no competing interest.

## Authors’ contributions

TI, NVS and LJT has organized the study, has collected, analysed and interpreted the data; TI has written the first version of manuscript; SD has analysed and interpreted the data and helped draft the paper; DA, SB, SS and MS have collected the data and edited the paper. All authors have read and approved the final manuscript.

## Supplementary Material

Additional file 1 Number of patients according to the number of vertebral fractures. Click here for file

Additional file 2 Distribution of patients according to fracture site. Click here for file
