# Supplementary material for: Validation of the osteoporosis quality of life questionnaire QUALEFFO-41 for the Serbian population
Source: Health Qual Life Outcomes. 2012 Jun 18;10:74. doi: 10.1186/1477-7525-10-74 (PMC3464801; doi:10.1186/1477-7525-10-74)
Supplement: Additional file 1 — Number of patients according to the number of vertebral fractures. [file 1477-7525-10-74-S1.pdf]

Number of patients according to the number of vertebral fractures

Number of fractures	Number of patients (%)
1	27 (55.10)
2	8 (16.33)
3	5 (10.20)
4	4 (8.16)
5	3 (6.12)
6	1 (2.04)
7	0 (0.00)
8	0 (0.00)
9	1(2.04)
