# Supplementary material for: Validation of the osteoporosis quality of life questionnaire QUALEFFO-41 for the Serbian population
Source: Health Qual Life Outcomes. 2012 Jun 18;10:74. doi: 10.1186/1477-7525-10-74 (PMC3464801; doi:10.1186/1477-7525-10-74)
Supplement: Additional file 2 — Distribution of patients according to fracture site. [file 1477-7525-10-74-S2.pdf]

Distribution of patients according to the fracture site

Fracture site	Number of patients (%)
L1	25 (24.04)
L2	11 (10.58)
L3	6 (5.77)
L4	10 (9.62)
L5	4 (3.85)
T5	1 (0.96)
T6	2 (1.92)
T7	8 (7.69)
T8	8 (7.69)
T9	6 (5.77)
T10	2 (1.92)
T11	9 (8.65)
T12	12 (11.54)
